# Post-Mortem Diagnosis of Pediatric Dengue Using Minimally Invasive Autopsy during the COVID-19 Pandemic in Brazil

**DOI:** 10.3390/tropicalmed7070123

**Published:** 2022-06-30

**Authors:** Deborah N. Melo, Giovanna R. P. Lima, Carolina G. Fernandes, André C. Teixeira, Joel B. Filho, Fernanda M. C. Araújo, Lia C. Araújo, André M. Siqueira, Luís A. B. G. Farias, Renata A. A. Monteiro, Jaume Ordi, Miguel J. Martinez, Paulo H. N. Saldiva, Luciano P. G. Cavalcanti

**Affiliations:** 1 Serviço de Verificação de Óbitos Dr Rocha Furtado, Fortaleza 60842-395, Brazil; deborahnmb@gmail.com (D.N.M.); andrect3@hotmail.com (A.C.T.); joelpatologista@gmail.com (J.B.F.); 2 Programa de Pós-graduação em Patologia, Universidade Federal do Ceará, Fortaleza 60020-181, Brazil; 3 Faculdade de Medicina, Centro Universitário Christus, Fortaleza 60190-180, Brazil; grolimlima@gmail.com (G.R.P.L.); carolfernandes.hp7@gmail.com (C.G.F.); 4 Argos Laboratory, Fortaleza 60175-047, Brazil; 5 Fundação Oswaldo Cruz, Eusebio 61760-000, Brazil; fernandamontenegrocaraujo@gmail.com; 6 Programa de Residencia Medica em Patologia pela Universidade Federal do Ceará, 60, Fortaleza 60020-181, Brazil; araujolc@gmail.com; 7 Fundação Oswaldo Cruz, Rio de Janeiro 21040-360, Brazil; amsiqueira@gmail.com; 8 Hospital São José de Doenças Infecciosas, Fortaleza 60455-610, Brazil; luisarthurbrasilk@hotmail.com; 9 Departamento de Patologia, Faculdade de Medicina da Universidade de São Paulo, São Paulo 01246-903, Brazil; reacademic@gmail.com (R.A.A.M.); pepino@usp.br (P.H.N.S.); 10 ISGlobal, Barcelona Institute for Global Health, Hospital Clínic, Universitat de Barcelona, 08036 Barcelona, Spain; jordi@clinic.cat; 11 ISGlobal, Hospital Clínic, University of Barcelona, 08007 Barcelona, Spain; myoldi@clinic.cat; 12 Programa de Pós-graduação em Saúde Coletiva, Universidade Federal do Ceará, Fortaleza 60020-181, Brazil

**Keywords:** severe dengue, autopsy, minimally invasive autopsy, arbovirus, COVID-19

## Abstract

We report the first pediatric disease in which the use of minimally invasive autopsy (MIA) confirmed severe dengue as the cause of death. During the COVID-19 pandemic, a previously healthy 10-year-old girl living in north-eastern Brazil presented fever, headache, diffuse abdominal pain, diarrhoea, and vomiting. On the fourth day, the clinical symptoms worsened and the patient died. An MIA was performed, and cores of brain, lungs, heart, liver, kidneys, and spleen were collected with 14G biopsy needles. Microscopic examination showed diffuse oedema and congestion, pulmonary intra-alveolar haemorrhage, small foci of midzonal necrosis in the liver, and tubular cell necrosis in the kidneys. Dengue virus RNA and NS1 antigen were detected in blood and cerebrospinal fluid samples. Clinical, pathological, and laboratory findings, in combination with the absence of other lesions and microorganisms, allowed concluding that the patient had died from complications of severe dengue.

## 1. Introduction

Dengue is the most important arbovirus worldwide, causing epidemics with a high human health and economic impact. Severe symptoms mainly affect the pediatric population from endemic low- and middle-income countries [1,2].

In Brazil, dengue remains the most widespread disease caused by arbovirus, even after the introduction of Zika and chikungunya. In north-eastern Brazil, deaths from dengue are frequent, even in non-epidemic years, especially in socially vulnerable populations [1,2,3]. Clinically, most dengue infections are either asymptomatic or produce mild disease [1,2,3]. However, given the high number of infections, severe cases are often reported during epidemics and represent a challenge for diagnosis and clinical management. In fatal cases, most organs and systems are affected, particularly the heart, central nervous system, gastrointestinal tract, and kidneys. 

After the establishment of Death Verification Services (DVS) in Brazil, the use of conventional autopsy (CA), the gold standard technique for the diagnosis of deaths caused by dengue, has contributed to the detection of patients clinically not diagnosed [4] and has significantly reduced neglected and underreported cases. However, the existence of few DVS in the cities, together with the low acceptability of CA among the relatives of the deceased and the lack of financial resources and specialised personnel, has resulted in limited implementation of this procedure [5]. Thus, the application of new strategies for post-mortem tissue collection is necessary, particularly for pediatric deaths, as rejection of CA by the relatives is very high in this population [5,6,7,8,9,10]. 

Minimally invasive autopsy (MIA) has been used as an alternative to CA with promising results [8,10,11,12,13,14]. This technique allows obtaining core biopsies of key organs by percutaneous puncture, with or without guidance with an imaging technique. MIA has been widely used in the context of the COVID-19 pandemic as a fast and non-disfiguring method with minimal biological risk for the personnel performing the procedure [8,9,15,16,17]. However, current knowledge of the performance of this technique for arboviral diseases in the paediatric population is very limited.

We report the first case of fatal dengue infection, which occurred in a previously healthy 10-year-old girl living in north-eastern Brazil during the COVID-19 pandemic. MIA sampling allowed correct diagnosis and showed complete agreement with the CA. We show that this acceptable, simplified, and non-disfiguring post-mortem technique can reliably diagnose death from severe dengue.

## 2. Case Report

A 10-year-old girl presented with fever, headache, diffuse abdominal pain, diarrhoea, and vomiting at the end of June 2021. She was previously healthy and had no comorbidities. A previously healthy 10-year-old girl with no comorbidities presented with fever, headache, diffuse abdominal pain, diarrhoea, and vomiting at the end of June 2021. The patient was initially treated with dipyrone. After 24 h, the patient presented dark stools. Two days later her clinical condition worsened and she was admitted to an emergency care unit (ECU), in which a blood count revealed thrombocytopenia (57,000/mm^3^). Intravenous hydration, antipyretics, and antiemetics were administered. After 3 days, the abdominal pain worsened, and the patient developed cutaneous pallor, arterial hypotension, and drowsiness, and was transferred to a paediatric hospital, where she arrived pale, with cold skin, thin pulse, gasping, dehydrated, and with tense abdomen. Myocarditis was considered by the physician. A femoral central venous access allowed expansion with albumin. The blood count revealed mild anaemia (haemoglobin 11.9 g/dL, haematocrit 35.9%), lymphopenia (92/mm^3^), and thrombocytopenia (57,000/mm^3^ on admission, which dropped to 20,000/mm^3^ within a few hours). Liver enzymes were above reference levels during hospitalisation (aspartate aminotransferase 741.1 U/L; alanine aminotransferase 248.9 U/L). She also had altered renal function, hyperkalaemia (10 mmol/L), and severe metabolic acidosis (pH 6.7). Activated partial thromboplastin time and prothrombin time were prolonged (Table 1). The next day, the patient suffered cardiorespiratory arrest, unresponsive to resuscitation measures. There was profuse bleeding through the oropharynx, trachea, and stomach. The clinical diagnoses were severe acute hepatitis of unexplained cause, acute renal dysfunction, and shock.

The mother of the patient reported the presence of several neighbors with similar symptoms and the recent admission of an aunt who lived with her, who had been diagnosed with severe dengue. Neither respiratory symptoms nor recent contact with suspected or confirmed cases of COVID-19 were described. Remarkably, co-circulation of SARS-CoV-2 and dengue has recently been reported in the Americas [18].

The body was sent to the DVS Dr Rocha Furtado (DVS-RF), where an MIA followed by CA were performed, after consent provided by the mother. The post-mortem procedures were performed as part of a study approved by the Research Ethics Committee through protocol CAAE 27162619.1.0000.5049, number 3,851,684.

Nasopharyngeal swabs were routinely tested by quantitative reverse transcriptase- polymerase chain reaction (qRT-PCR) for SARS-CoV-2 in all DVS-RF autopsies performed during the COVID-19 pandemic. About 20 mL of blood and 2 mL of cerebrospinal fluid (CSF) were collected as part of the MIA procedure before the CA. The two post-mortem procedures were analysed by two different pathologists.

For the MIA, 20 cm, 14 Gauge percutaneous biopsy needles were used. Four brain cores (1.2 to 1.3 cm) were obtained by introducing the biopsy needle through the right and left nasal cavity, piercing the cribriform plate of the sphenoid bone. The right and the left lungs were punctured between the third and fourth intercostal spaces, and four cores from each lung (0.5 to 0.6 cm from the right lung; 1.2 to 1.4 cm from left lung) were collected. Four cores (0.8 to 0.9 cm) were obtained from the heart, after puncture in the fifth intercostal space. The liver was punctured in the right 11th intercostal space, in the anterior axillary line, and four tissue cores (0.7 to 1.0 cm) were obtained. Punctures directed to the kidneys were performed in the right and left subcostal spaces and four tissue fragments from each side were obtained (1.5 to 1.7 cm right, 1.0 to 1.2 cm left). Finally, four cores were collected from the splenic area (0.8 to 1.0 cm). 

CA was performed following the DVS-RF protocol (4), after opening all cavities. The brain was swollen (weight 1310 g). Bilateral pleural effusion and ascites were observed. The lungs were oedematous and showed areas of haemorrhage (weight: 365 g left and 375 g right). The liver and the spleen showed congestion and weighed 1200 g and 415 g, respectively. One-hundred-and-fifty milliliters of fresh blood were identified in the stomach. The kidneys were pale and oedematous (weight: 110 g right and 100 g left). The adrenals showed no abnormalities.

Microscopic examination showed oedema and congestion in all organs, foci of intra-alveolar haemorrhage in the lungs, and foci of midzonal necrosis in the liver. Hypoplasia of the white pulp of the spleen was associated with abundant macrophages with large clear nuclei. There was extensive coagulative necrosis of the cortical tubules of the right kidney.

Samples of the left kidney and spleen of the MIA showed only skeletal muscle, connective tissue, vessels, and nerves under microscopy, with no cores of parenchyma.

The nasopharyngeal swab, blood, and CSF were sent to the Central Laboratory of Public Health of Ceará (LACEN-CE) for laboratory tests: qRT-PCR for respiratory viruses, arboviruses (dengue, Zika, and chikungunya), and detection of dengue NS1 antigen in blood and CSF [19]. A blood culture for bacterial research was also performed.

**Figure 1:**
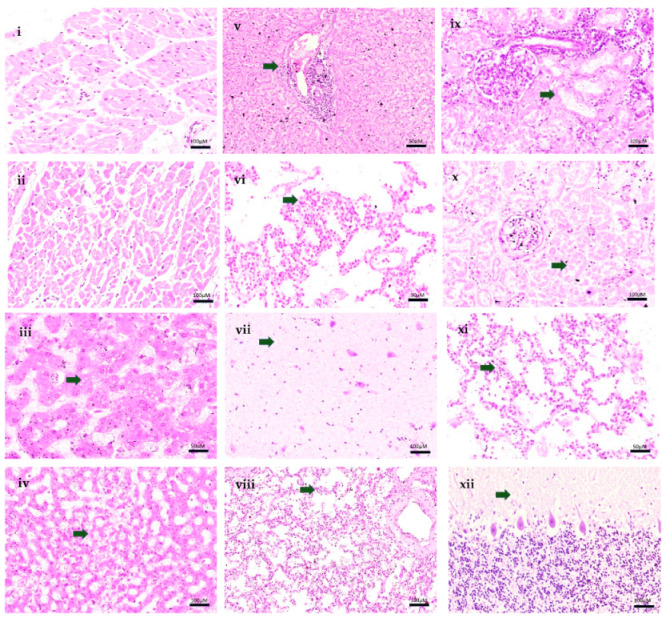
Images of MIA and CA samples of patient. (**i**)—Heart. Cardiac muscle fibres. (HE, 100X—MIA). (**ii**)—Heart. Cardiac muscle fibres. (HE, 100X—CA). (**iii**)—Liver. Mild microvesicular steatosis. (HE, 50X—MIA). (**iv**)—Liver. Midzonal necrosis of hepatocytes. (HE, 100X—MIA). (**v**)—Liver. Mononuclear portal infiltrate. Edema and congestion. (HE, 50X)—CA). (**vi**)—Lung. Interstitial pneumonitis. (HE, 50X—MIA). (**vii**)—Lung. Interstitial pneumonitis. (HE, 100X—CA). (**viii**)—Lung. Interstitial pneumonitis. Alveolar edema. (HE, 100X—CA). (**ix**)—Kidney R. Necrosis of renal tubules. (HE, 100X—MIA). (**x**)—Kidney Necrosis of renal tubules. (HE, 100X—CA). (**xi**)—Brain. Brain edema. (HE, 50X—MIA). (**xii**)—Cerebellum. Edema. (HE, 100X—CA). Legend: CA = conventional autopsy; MIA = minimally invasive autopsy.

The following findings were of note: midzonal hepatocyte necrosis with rare acidophilic bodies seen only in the MIA samples, which were better preserved; enlargement of the alveolar septa by inflammatory cells (viral interstitial pneumonitis), edema and foci of intraalveolar hemorrhage seen in both MIA and CA; and acute tubular necrosis in the kidneys (Figure 1). Previous studies reporting histological findings in fatal cases of dengue have reported similar changes, including diffuse congestion and hemorrhage, alveolar edema, and liver cell necrosis [20]. 

The nasopharyngeal swab sample tested negative for SARS-CoV-2 RNA and there was no microbial growth in the blood culture. The qRT-PCR test for arboviruses identified the presence of DENV-2 RNA in the blood sample, and the NS1 antigen (kit J. Mitra & Co. Pvt. Ltd.) tests were positive for dengue in the blood and CSF samples [21] All tests performed for Zika and chikungunya viruses were negative (Table 1).

Clinical features, such as upper digestive and pulmonary hemorrhage, acute tubular necrosis, and shock causing death, in conjunction with the pathological and laboratory findings, were in keeping with the diagnosis of death due to complications of severe dengue. Remarkably, the samples collected by the MIA in this pediatric patient were sufficient to confirm the diagnosis of severe dengue and were completely in agreement with the samples collected by the CA (Figure 1). 

## 3. Conclusions

Disease surveillance and patient healthcare requires adequate ascertainment of the cause of death, especially in the current context of circulation of multiple arboviruses and other pathogens with the potential of causing epidemics. In a scenario of reduced acceptability of CA, MIA is a promising tool, which has proven to be successful even during the COVID-19 pandemic, for diagnosing arboviral-related deaths.

## Figures and Tables

**Table 1:** Results of laboratory tests.

Exam	27 June 2021 (3 Days of Symptom)	28 June 2021 (4 Days of Symptom)	Reference Values
Red Cells	4.29 million/mm^3^	4.11 million/mm^3^	4.1 to 5.3 million/mm^3^
Haemoglobin	12.3 g/dL	11.9 g/dL	12 to 14.5 g/dL
Haematocrit	35.9%	35%	36 to 43%
Leukocytes	2300/mm^3^	5600/mm^3^	3400 to 10,800/mm^3^
Neutrophils	1955/mm^3^	4424/mm^3^	1500 to 8500/mm^3^
Rod Neutrophils	69/mm^3^	224/mm^3^	0 to 860/mm^3^
Segmented Neutrophils	1886/mm^3^	4200/mm^3^	1500 to 8500/mm^3^
Eosinophils	0/mm^3^	56/mm^3^	0 to 500/mm^3^
Lymphocytes	92/mm^3^	672/mm^3^	1500 to 6500/mm^3^
Monocytes	253/mm^3^	336/mm^3^	0 to 800/mm^3^
Basophils	0/mm^3^	0/mm^3^	0 to 200/mm^3^
Platelets	57,000/mm^3^	20,000/mm^3^	150 to 450 mil/mm^3^
Atypical Lymphocytes	-	112/mm^3^	0%
MPV	8.3 fL	7.5 fL	9.2 to 12.6 fL
Ultrasensitive C-reactive protein	2.96 mg/dL	2.37 mg/dL	<0.10 mg/dL
Magnesium	-	2.02 mg/dL	2.02 to 2.75 mg/dL
Potassium	4.0 mmol/L	6.8 mmol/L	3.5 to 5.1 mmol/L
Sodium	136 mmol/L	139 mmol/L	136 to 145 mmol/L
AST	83.3 U/L	741.1 U/L	17 to 33 U/L
ALT	27.3 U/L	248.9 U/L	9 to 23 U/L
Urinary Urobilinogen	3.0 mg/dL	-	< 1.0 mg/dL
Creatinine	-	0.82 mg/dL	0.32 to 0.61 mg/dL
Urea	-	28.9 mg/dL	19.2 to 46.2 mg/dL
TAP—prothrombin time	-	16.8 s	9.4 to 12.5 s
APTT- activated partial thromboplastin	-	49.8 s	25.1 to 36.5 s
Laboratory tests performed after death			
Blood culture RT-PCR for SARS-CoV-2 qRT-PCR for dengue qRT-PCR for Zika qRT-PCR for chikungunya NS1 antigen			No microbial growth Not Detectable Positive Negative Negative Positive

Subtitle: MPV—mean platelet volume, AST—aspartate aminotransferase, ALT—alanine aminotransferase, TAP—prothrombin time, APTT—activated partial thromboplastin, RT-PCR—reverse transcriptase-polymerase chain reaction, qRT-PCR—quantitative reverse transcriptase-polymerase chain reaction.

## Data Availability

Not applicable.
